# THE ROLE OF GERONTOLOGISTS IN CONTRIBUTING TOWARD PUBLIC POLICY TO ACHIEVE THE UN’S SUSTAINABLE DEVELOPMENT GOALS

**DOI:** 10.1093/geroni/igz038.297

**Published:** 2019-11-08

**Authors:** Ruth Finkelstein

**Affiliations:** The Brookdale Center on Aging, Hunter College, New York City, United States

## Abstract

The UN SDGs are aspirational goals whose achievement requires intentional policy-making at each level of government. Advocates for older people argued for goals specifically addressing older people, the fastest growing segment of the world’s population. While unsuccessful, we did achieve a targets monitoring template that reports outcomes separately by age. This innovation will bring helpful attention to generational differences in needs and progress, but gerontologists must play a critical role for this innovation to be realized as a benefit. Gerontologists must actively prevent SDG implementation for older people from being siloed into aging services departments. Aging services agencies do not have the resources or skills to achieve the SDGs – they do not control policy levers to end poverty, ensure gender equity or sustainable urban development. Yet, too often, once a population is separately counted, responsibility for its wellbeing follows. Gerontologists must help governments adopt an “age in everything” lens.

